# Probing stereoselective inhibition of the acyl binding site of cholesterol esterase with four diastereomers of 2'-*N*-α-methylbenzylcarbamyl-1, 1'-bi-2-naphthol

**DOI:** 10.1186/1471-2091-6-17

**Published:** 2005-09-22

**Authors:** Shyh-Ying Chiou, Cheng-Yue Lai, Long-Yau Lin, Gialih Lin

**Affiliations:** 1 Institute of Medicine and Department of Neurosurgery, Chung Shan Medical University, Taichung 402, Taiwan; 2 Department of Chemistry, National Chung-Hsing University, Taichung 402, Taiwan

## Abstract

**Background:**

Recently there has been increased interest in pancreatic cholesterol esterase due to correlation between enzymatic activity in vivo and absorption of dietary cholesterol. Cholesterol esterase plays a role in digestive lipid absorption in the upper intestinal tract, though its role in cholesterol absorption in particular is controversial. Serine lipases, acetylcholinesterase, butyrylcholinesterase, and cholesterol esterase belong to a large family of proteins called the α/β-hydrolase fold, and they share the same catalytic machinery as serine proteases in that they have an active site serine residue which, with a histidine and an aspartic or glutamic acid, forms a catalytic triad. The aim of this work is to study the stereoselectivity of the acyl chain binding site of the enzyme for four diastereomers of an inhibitor.

**Results:**

Four diastereomers of 2'-*N*-α-methylbenzylcarbamyl-1, 1'-bi-2-naphthol (**1**) are synthesized from the condensation of R-(+)- or S-(-)-1, 1'-bi-2-naphthanol with R-(+)- or S-(-)-α-methylbenzyl isocyanate in the presence of a catalytic amount of pyridine in CH_2_Cl_2_. The [α]^25^_D _values for (1R, αR)-**1**, (1R, αS)-**1**, (1S, αR)-**1**, and (1S, αS)-**1 **are +40, +21, -21, and -41°, respectively. All four diastereomers of inhibitors are characterized as pseudo substrate inhibitors of pancreatic cholesterol esterase. Values of the inhibition constant (*K*_*i*_), the carbamylation constant (*k*_2_), and the bimolecular rate constant (*k*_*i*_) for these four diastereomeric inhibitors are investigated. The inhibitory potencies for these four diastereomers are in the descending order of (1R, αR)-**1**, (1R, αS)-**1**, (1S, αR)-**1**, and (1S, αS)-**1**. The *k*_2 _values for these four diastereomers are about the same. The enzyme stereoselectivity for the 1, 1'-bi-2-naphthyl moiety of the inhibitors (R > S, ca. 10 times) is the same as that for 2'-N-butylcarbamyl-1, 1'-bi-2-naphthol (**2**). The enzyme stereoselectivity for the α-methylbenzylcarbamyl moiety of the inhibitors is also R > S (2–3 times) due to the constraints in the acyl binding site.

**Conclusion:**

We are the first to report that the acyl chain binding site of cholesterol esterase shows stereoselectivity for the four diastereomers of **1**.

## Background

Recently there has been increased interest in pancreatic cholesterol esterase (CEase, EC 3.1.1.13) due to correlation between enzymatic activity in vivo and absorption of dietary cholesterol [1,2]. Physiological substrates include cholesteryl esters, retinyl esters, triacylglycerols, vitamin esters, and phospholipids [3-5]. CEase plays a role in digestive lipid absorption in the upper intestinal tract, though its role in cholesterol absorption in particular is controversial [1,6]. A recent report indicates that CEase is directly involved in lipoprotein metabolism, in that the enzyme catalyzes the conversion of large LDL to smaller, denser, more cholesteryl ester-rich lipoproteins, and that the enzyme may regulate serum cholesterol levels [7,8]. Serine lipases, acetylcholinesterase, butyrylcholinesterase, and CEase belong to a large family of proteins called the α/β-hydrolase fold [9,10], and they share the same catalytic machinery as serine proteases in that they have an active site serine residue which, with a histidine and an aspartic or glutamic acid, forms a catalytic triad [11,12]. The conservation of this catalytic triad suggests that as well as sharing a common mechanism for substrate hydrolysis, that is, formation of a discrete acyl enzyme species via the active site serine hydroxy group, serine proteases, CEase, and lipases may well be expected to be inhibited by the same classes of mechanism-based inhibitors such as phosphorothiolates [13], pyrones [14], fluoroketones [15], boronic acids [16], and carbamates [16-29].

**Figure 1:**
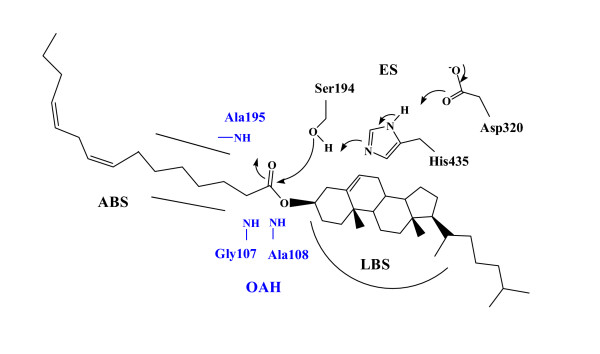
Possible interactions for cholesteryl linoleate in the active site of CEase [30,31].

The crystal structure of the active site region of pancreatic CEase [30,31] is similar to *Torpedo californica *acetylcholinesterase (AChE) [32], *Candida rugosa *lipase (CRL) [33,34], *Geotrichum candidum *lipase (GCL) [35], and *Pseudomonas *species lipase (PSL) [36,37]. Moreover, the active site of CEase like CRL, GCL, PSL, and acetylcholinesterase may consist of at least five major binding sites (Figure 1) [23,24,30,31]: (a) an acyl chain binding site (ABS) that binds to the acyl chain of the substrate and is opened by the removal of C-terminal 574–579 in which is bent in shape and contains a deep, wide hole from the evacuation of Phe579, (b) an oxyanion hole (OAH), the H-bonding peptide NH functions of Gly107, Ala108, and Ala195, that stabilizes the tetrahedral species, (c) an esteratic site or the catalytic triad (ES), comprised of Ser194-His435-Asp320, that is involved in nucleophilic attack to the substrate carbonyl group and in general acid-base catalysis, and (d) a leaving group binding site (LBS) or/and the second alkyl chain or group binding site (SACS) that binds to the cholesterol part of cholesterol ester or the second fatty acid chain of triacylglycerol and is located at the opposite direction of ABS.

**Figure 2:**
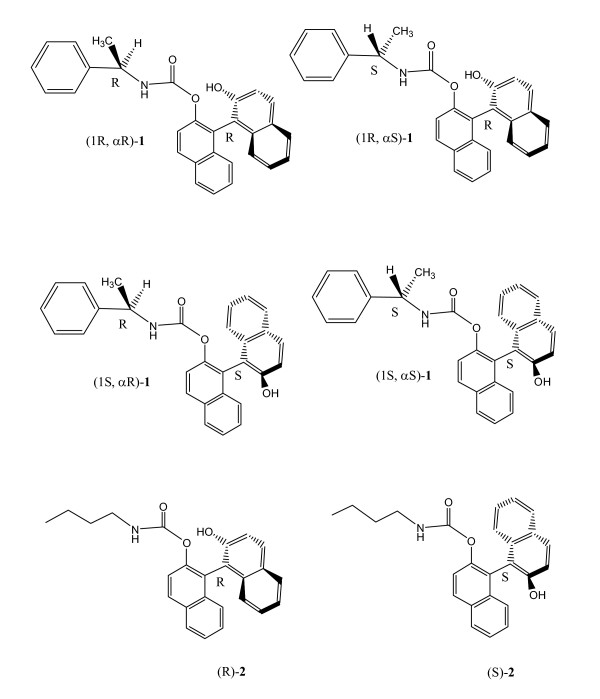
Structures of the four diastereomers of carbamates **1 **and the two atropisomers of **2**.

Previous work has shown that CEase is stereoselectively inhibited by the two atropisomers (or enantiomers) of 1, 1-bi-2-naphtyl carbamates due to the stereoselective binding at LBS of the enzyme [20,22]. Doorn et al. have also reported that CEase is stereoselectively inhibited by the four diastereomers of isomalathion due to stereoselectivity for both ES and LBS of the enzyme [13]. The aim of this study is to extend the stereoselectivity to the four diastereomers of inhibitors by adding two extra bonds between a chiral center and (or a chiral axis of the inhibitors. In other words, we may probe the double selectivity for both ABS and LBS of the enzyme. Thus, four diastereomers of 2'-*N*-α-methylbenzylcarbamyl-1, 1'-bi-2-naphthol (**1**), 2'-*N*-(R)-α-methylbenzylcarbamyl-(R)-1, 1'-bi-2-naphthol ((1R, αR)-**1**), 2'-*N*-(S)-α-methylbenzylcarbamyl-(R)-1, 1'-bi-2-naphthol ((1R, αS)-**1**), 2'-*N*-(R)-α-methylbenzylcarbamyl-(S)-1, 1'-bi-2-naphthol ((1S, αR)-**1**), and 2'-*N*-(S)-α-methylbenzylcarbamyl-(S)-1, 1'-bi-2-naphthol ((1S, αS)-**1**) (Figure 2), are synthesized from condensation of (R)- or (S)-1, 1'-bi-2-naphthol with (R)-or (S)-α-methylbenzyl isocyanate in the presence of pyridine in dichloromethane. The stereoselectivity of CEase inhibition by the four diastereomers of **1 **is evaluated kinetically.

**Figure 3:**
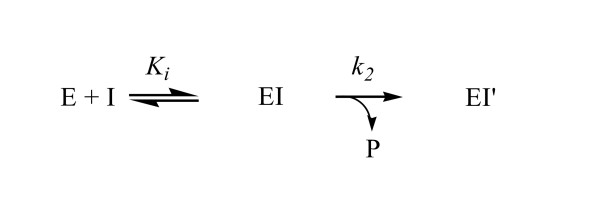
Kinetic scheme for the pseudo substrate inhibition of CEase.

Most carbamate inhibitors are characterized as the pseudo substrate inhibitors of CEase (Figure 3) [16-29] and meet some of the criteria proposed by Abeles and Maycock [38]. First, the inhibition is time-dependent and follows pseudo-first-order kinetics; second, with increasing concentration of inhibitor the enzyme displays saturation kinetics; third, the enzyme is protected from inhibitions by carbamate by binding of a competitive inhibitor such as trifluoroacetophenone (TFA). The *K*_*i *_step leads to the tetrahedral intermediate and the *k*_2 _step leads to the carbamyl enzyme intermediate. Moreover, values of *K*_*i *_and *k*_2 _can be calculated from Equation 1 [16-29]:

*k*_*app *_= *k*_2 _[I]/(*K*_*i*_(1+ [S]/*K*_*m*_)+ [I]) (1)

In Equation 1, *k*_app _values are first-order rate constants which can be obtained as described in Hosie et al. [17]. Bimolecular rate constant, *k*_*i *_= *k*_2_/*K*_*i*_, is related to overall inhibitory potency.

## Results

For the first time, we synthesize four optical pure diastereomers of **1**. (1R, αR)-**1**, (1R, αS)-**1**, (1S,αR)-**1**, and (1S, αS)-**1 **(Figure 2) are synthesized from the condensation of R-(+)- or S-(-)-1, 1'-bi-2-naphthanol with R-(+)- or S-(-)-α-methylbenzyl isocyanate in the presence of a catalytic amount of pyridine in CH_2_Cl_2_. The [α]^25 ^_*D*_values for (1R, αR)-**1**, (1R, αS)-**1**, (1S,αR)-**1**, and (1S, αS)-**1 **are +40, +21, -21, and -41°, respectively.

**Figure 4:**
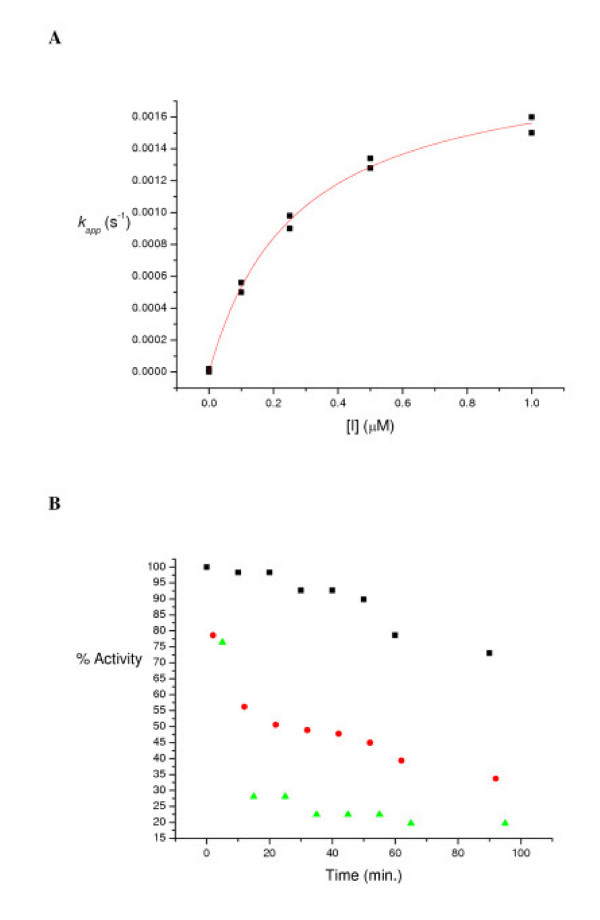
A: The *k*_*app *_vs. [I] plot for inhibition of the CEase-catalyzed hydrolysis of PNPB by (1R, αR)-**1**. [PNPB] = 50 μM. The solid line is a least-squares fit to Eq. (1) [17]; the parameters of the fit are *K*_*i *_= 0.27 ± 0.01 μM and *k*_2 _= (2.0 ± 0.2) × 10^-3 ^s^-1^. B: % activity of CEase vs. the time period for inhibition of the enzyme with (1R, αR)-**1 **(50 nM) in the absence and presence of TFA (2 μM). [PNPB] = 50 μM. All the procedures followed those of Hosie et al. [17]. For the control experiments (squares), CEase was incubated alone at 25.0°C for a period of time before the inhibition reaction (CEase + PNPB + (1R, αR)-**1**). For the carbamate inhibition experiments (triangles), CEase was incubated with (1R, αR)-**1 **(50 nM) at 25.0°C for a period of time before the enzyme reaction (CEase + PNPB). For the protection experiments (circles), CEase was incubated with (1R, αR)-**1 **(50 nM) and TFA (2 μM) at 25.0°C for a period of time before the enzyme reaction (CEase + PNPB).

Like most carbamates, the four diastereomers of **1 **are characterized as the pseudo substrate inhibitors of CEase (Figures 3 and 4) and meet some of the criteria proposed by Abeles and Maycock [38]. When CEase is incubated with a carbamate in the presence of TFA (2 μM), a known competitive inhibitor of CEase [22] before the inhibition reaction, the enzyme is protected from inhibition by carbamate by binding of TFA as described in Hosie et al. [17] (Figure 4B).

The inhibition data for CEase by the four diastereomers of **1 **and the two enantiomers of **2 **are summarized (Table 1). The stereochemical preference of CEase for the binaphthyl moiety of **1 **(R > S, ca. 10 times) is the same as that for **2 **[20,22]. The stereoselectivity of CEase for the α-methylbenzyl moiety of **1 **is also the R-form (2–3 times over S-form).

**Table 1:** Inhibition constants for CEase-catalyzed hydrolysis of PNPB in the presence of the four diastereomers of 1 and the two enantiomers of 2

Inhibitor	K_i_(μM)	k_2_(10^-3^s^-1^)	k_i_(10^3 ^M^-1^s^-1^)
(1R, αR)**-1**	0.20 ± 0.01	2.0 ± 0.2	10 ± 1
(1R, gαS)**-1**	0.50 ± 0.03	2.0 ± 0.2	4.0 ± 0.4
(1S, gαR)**-1**	2.0 ± 0.1	2.0 ± 0.2	1.0 ± 0.1
(1S, gαS)**-1**	6.0 ± 0.4	1.8 ± 0.2	0.30 ± 0.03
(R)**-2**^a^	0.8 ± 0.1	10 ± 1	12 ± 2
(S)**-2**^a^	1.3 ± 0.1	6.0 ± 0.5	5.0 ± 0.6

^a^Taken from references [20,22].

Among the four diastereomers of **1**, (1R, αR)-**1 **is the most potent inhibitor and its overall inhibitory potency (*k*_*i*_) is about the same as that of R-**2 **(Table 1). On the other hand, (1S, αS)-**1 **is the least potent inhibitor of CEase and its overall inhibitory potency is about 17-fold lower than that of S-**2**. All *k*_2 _values for the CEase inhibition by**1 **are about the same (Table 1).

## Discussion

**Figure 5:**
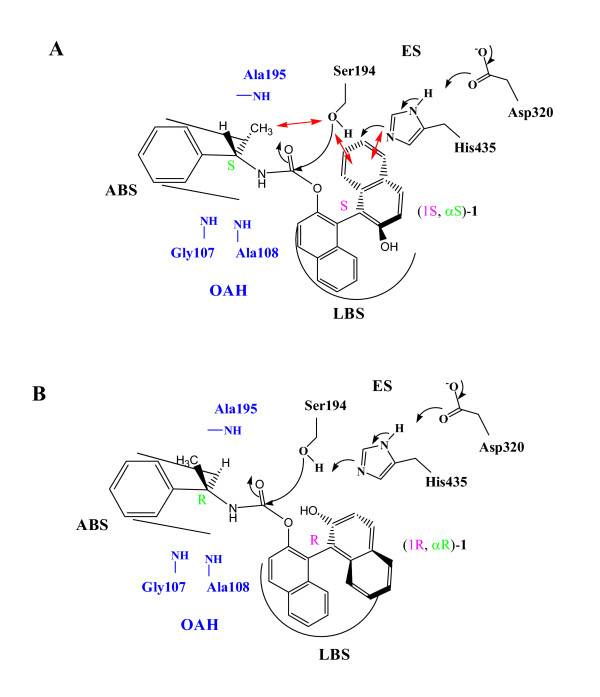
Possible interactions between the stereoisomers of **1 **and CEase [31,31]. (A) CEase and (1S, αS)-**1**. The methyl benzyl moiety of the inhibitor binds to ABS of the enzyme. Three unfavorable repulsions (in red) from the methyl moiety and Ser194, the naphthyl moiety and Ser194, and the naphthyl moiety and His435 hinder the nucleophilic attack of Ser194 to the carbonyl group of the inhibitor. (B) CEase and (1R, αR)-**1**. There is no unfavorable repulsion for the nucleophilic attack of Ser194 to the carbonyl group of the inhibitor.

According to the X-ray crystal structure, CEase-catalyzed hydrolysis of cholesteryl linoleate has been proposed (Figure 1) [30,31]. Like most carbamates, the four diastereomers of **1 **are characterized as the pseudo substrate inhibitors of CEase (Figures 3 and 4) [16-29] and meet some of the criteria proposed by Abeles and Maycock [38]. Therefore, the CEase inhibition by the four diastereomers of **1**is proposed (Figure 5) [4]. In this mechanism, the α-methylbenzylcarbamyl moiety of **1 **is proposed to bind to ABS of the enzyme, and the binaphthyl moiety of **1 **is proposed to bind to LBS of the enzyme. The stereochemical preference of CEase for the binaphthyl moiety of **1 **(1R > 1S in Table 1) at LBS of the enzyme is therefore identical to that of **2 **(R > S) due to the fact that the nucleophilic attack of the Ser194 of the enzyme to the carbonyl group of the inhibitor sterically hinder from one of the naphthyl group of the inhibitors (Figure 5A) [20,22]. Since 4-nitrophenyl-*N*-benzyl-carbamate is a very potent pseudo substrate inhibitor of CEase [21,25,26], the benzylcarbamyl moiety of the inhibitor is believed to bind tightly to ABS of the enzyme. Similarly, the α-methylbenzylcarbamyl moiety of **1 **is also believed to bind to ABS of the enzyme. The stereochemical preference of CEase for the α-methylbenzylcarbamyl moiety of **1 **at ABS of the enzyme is also R > S (αR > αS in Table 1). The possible reason for this is the fact that one of the naphthyl group and the α-methyl group of (1S, αS)-**1 **are located at the same side of the nucleophilic attack of Ser194 when the inhibitor binds to CEase and therefore these two groups of the inhibitor sterically hinder the nucleophilic attack of Ser194 to the inhibitor (Figure 5A). On the other hand, (1R, αR)-**1 **does not have any hindrance for the nucleophilic attack of Ser194 (Figure 5B) and therefore (1R, αR)-**1 **is the most potent inhibitor among the four diastereomers of **1 **(Table 1).

The stereoselectivity of CEase at ABS of the enzyme for the α-methylbenzyl group of **1 **(R > S) (Table 1) is the same as that of CRL at its ABS for 2-methyl-6-(2-thienyl) hexanate [39]. For the *K*_*i *_step (Figure 3), (1R, αR)-**1 **and (1S, αR)-**1 **bind to CEase 2.5 and 3 times more tightly than (1R, αS)-1 and (1S, αS)-1, respectively. The *K*_*i *_value with regard to the chiral center at the α-position of **1 **is quite low compared to that with regard to the binaphthol chiral axis of **1 **(Table 1) [20,22] and to that with regard to the phosphorus chiral center of isomalathion [13]. Therefore, we propose that ABS of CEase does not show high selectivity for the chiral acyl group due to a narrow and hydrophobic binding pocket for ABS [30,31], which selectively and tightly binds to the benzyl phenyl moiety of the inhibitor and results in the discrimination of stereoselectivity by either the hydrogen atom or the methyl group at the α-position of the four diastereomers of **1 **(Figure 5).

(1R, αR)-**1 **and (1R, αS)-**1 **are bound to CEase 10 and 12 times more tightly than (1S, αR)-1 and (1S, αS)-1, respectively (Table 1); however, R-**2 **is bound to CEase only 1.6 times more tightly than S-**2 **[20,22]. The possible reason is that the binding of the phenyl moiety of the α-methylbenzylcarbamyl group of **1 **to ABS (Figure 5) constrains the binaphthol moiety of **1 **to a more favorable conformation to bind with LBS, on the other hand, the *n*-butyl carbamyl of **2 **has lots of room to "breathe" in ABS and therefore the binaphthol moiety of**2 **has many conformations and results in loosely binding to LBS.

The *k*_2 _values for the four diastereomers of **1 **are about the same. This means that the *k*_2 _step is insensitive to the stereochemistry of **1**. In other words, the stereoselectivity of CEase for (1R, αR)-**1 **primarily results from the *K*_*i *_step. The *k*_2 _values for all diastereomers of**1 **are lower than those for the two atropisomers of **2 **(Table 1). The possible reason is that the *n*-butylcarbamyl enzyme from both atropisomers of **2 **is relatively more stable than the α-methylbenzylcarbamyl enzymes from the four diastereomers of **1**.

Overall, we report that CEase has two stereoselective binding sites at LBS and ABS for the four diastereomers of **1**. CEase [13], *Chromobacterium viscosum *lipase, and *Rhizopus oryzal *lipase [40] also show two stereoselective binding sites at LBS and ES for organic phosphorus compounds. Therefore, it is possible that CEase and lipase may contain totally three stereoselective binding sites at ABS, ES, and LBS for the six diastereomers of substrates or inhibitors.

## Conclusion

Four diastereomers of **1 **are synthesized and characterized as the pseudo substrate inhibitors of pancreatic cholesterol esterase. The inhibitory potencies for these four diastereomeric inhibitors are in the descending order of (1R, αR)-**1**, (1R, αS)-**1**, (1S, αR)-**1**, and (1S, αS)-**1**. The enzyme stereospecificity toward the 1, 1'-bi-2-naphthyl moiety of the inhibitors is the R-form and is the same as that for **2**. The enzyme stereospecificity toward the α-methylbenzylcarbamyl moiety of the inhibitors is also R-form. For the first time, we observe that the acyl binding site of cholesterol esterase shows stereospecificity for diastereomeric inhibitors.

## Methods

### Materials

Porcine pancreatic CEase (ca. 70% pure since the observed *K*_*m *_value for this enzyme catalyzed hydrolysis of PNPB is 1.4 times higher than that for the pure enzyme [17]) and PNPB were obtained from Sigma; TFA and other chemicals were obtained from Aldrich. Silica gel used in liquid chromatography (Licorpre Silica 60, 200–400 mesh), medium pressure liquid chromatography column (LiChroprep Si 60) and thin layer chromatography plates (Kieselgel 60 F254) were obtained from Merck. An UV lamp as well as an UV detector (Linear UV-106 or ISCO UA-6) was used in detection. Hexane-ethyl acetate solvent gradient was used in liquid chromatography and medium pressure liquid chromatography. Other chemicals were of the highest quality available commercially. Carbamates **2 **were synthesized as described before [20,22].

### Instrumental methods

^1^H and ^13^C NMR spectra were recorded at 300 and 75.4 MHz (Varian-VXR 300 spectrometer), respectively. The ^1^H and ^13^C NMR chemical shifts were referred to internal Me_4_Si. UV spectra were recorded on an UV-visible spectrophotometer (Hewlett Packard 8452A or Beckman DU-650) with a cell holder circulated with a water bath. High resolution mass spectra were recorded at 70 eV on a Joel JMS-SX/SX-102A mass spectrophotometer. Elemental analyses were preformed on a Heraeus instrument.

### Synthesis of four diastereomers of **1**

(1R, αR)-**1**, (1R, αS)-**1**, (1S, αR)-**1**, and (1S, αS)-**1 **(Figure 2) were prepared from the condensation of R-(+)- or S-(-)-α-methylbenzyl isocyanate ([α]^20 ^_*D *_= +10° or -10°) with 1 equivalent of R-(+)- or S-(-)-1, 1'-bi-2-naphthol ([α]^20 ^_*D *_= +34° or -34°) in the presence of a catalytic amount of pyridine in CH_2_Cl_2 _at 25°C for 24 h (80–95 % yield). All products were purified by liquid chromatography or medium pressure liquid chromatography (silica gel, hexane-ethyl acetate) and characterized by ^1^H and ^13^C NMR spectra and high resolution mass spectra.

(1R, αR)-**1**, (1R, αS)-**1**, (1S, αR)-**1**, and (1S, αS)-**1**: ^1^H NMR (CDCl_3_, 300 MHz) δ/ppm 1.02 (d, J = 6.6 Hz, 3H, CH(Ph)CH_3_), 4.48 (quintet, J = 7 Hz, 1H, CH(Ph)CH_3_), 5.27 (d, J = 8.1 Hz, 1H, NH), 7.07–8.06 (m, 17H, aromatic H); ^13^C NMR (CDCl_3_, 75.4 MHz) δ/ppm 21.88 (CH3), 50.36 (CH(Ph)CH_3_), 122.45, 123.51, 125.43, 125.69, 126.08, 126.48, 126.60, 127.10, 127.24, 127.91, 128.18, 128.37, 128.53, 129.40, 131.40, 133.30, 133.41, 142.98, and 147.20 (aromatic Cs), 153.91 (C = O); High resolution mass spectra: Found: 433.1674; C_29_H_23_NO_3 _requires 433.1678. [α]^25 ^_*D *_= +40, +21, -21, and -41° for (1R, αR)-**1**, (1R, αS)-**1**, (1S, αR)-**1**, and (1S, αS)-**1**, respectively. The stability of these compounds is very high at -20°C (no significant change for the optical rotation in 1 month).

### Enzyme kinetics and data reduction

All kinetic data were obtained by using an UV-visible spectrophotometer that was interfaced to a computer. Microcal Origin (version 6.0) was used for all least squares curve fittings. The CEase inhibition was assayed as described in Hosie et al. [17]. The temperature was maintained at 25.0°C by a refrigerated circulating water bath. All reactions were performed in sodium phosphate buffer (pH 7.0) containing NaCl (0.1 M), acetonitrile (2% by volume), substrate PNPB (50 μM), triton X-100 (0.5 % by weight) and varying concentration of inhibitors (from 0.1 to 10 μM). The *K*_*m *_value for CEase-catalyzed hydrolysis of PNPB was calculated to be 140 ± 10 μM from the Michaelis-Menten equation. Requisite volumes of stock solution of substrate and inhibitors in acetonitrile were injected into reaction buffers via a pipet. CEase was dissolved in sodium phosphate buffer (0.1 M, pH 7.0). Reactions were initiated by injecting enzyme and monitored at 410 nm on the UV-visible spectrometer. First-order rate constants (the *k*_app _values) for inhibition of CEase were determined as described by Hosie et al. [17] Values of *K*_*i *_and *k*_2 _can be obtained by the parameters of non-linear least squares curve fittings of *k*_app _vs. [I] plot to Equation (1) (Figure 4A). Duplicate sets of data were collected for each inhibitor concentration.

## List of abbreviations used

ABS, acyl chain binding site; AChE, acetylcholinesterase, BChE, butyrylcholinesterase; CEase, cholesterol esterase; CRL, *Candida rugosa *lipase; ES, catalytic or esteratic site; GCL, *Geotrichum candidum *lipase; *k*_app_, first-order rate constants; *k*_2_, carbamylation constants; *k*_*i*_, bimolecular rate constant; LHIS, leaving group hydrophilic binding site; LBS, leaving group binding site; 2'-*N*-(R)-α-methylbenzylcarbamyl-(R)-1, 1'-bi-2-naphthol ((1R, αR)-**1**); 2'-*N*-(S)-α-methylbenzylcarbamyl-(R)-1, 1'-bi-2-naphthol ((1R, αS)-**1**); 2'-*N*-(R)-α-methylbenzylcarbamyl-(S)-1, 1'-bi-2-naphthol ((1S, αR)-**1**); 2'-*N*-(S)-α-methylbenzylcarbamyl-(S)-1, 1'-bi-2-naphthol ((1S, αS)-**1**); OAH, the oxyanion hole; PSL, *Pseudomonas *species lipase; PNPB, *p*-nitrophenyl butyrate; PSL, *Pseudomonas *species lipase; SACS, the second acyl chain binding site; TFA, trifluoroacetophenone.

## Authors' contributions

SYC carried out the enzyme kinetic studies. CYL participate in the synthesis of 4 diastereomers of carbamate inhibitors. LYL participated in the design of some parts of the study. GL drafted the manuscript and designed most parts of the study. All authors read and approved the final manuscript.
